# Cleared for Launch: Pre-flight Medical Assessment in the Commercial Spaceflight Era

**DOI:** 10.7759/cureus.109824

**Published:** 2026-05-28

**Authors:** Piercarlo Minoretti

**Affiliations:** 1 Occupational Health, Studio Minoretti, Oggiono, ITA

**Keywords:** aerospace medicine, civilian astronaut, commercial spaceflight, fitness to fly, pre-flight medical evaluation, space flight, spaceflight-associated neuro-ocular syndrome

## Abstract

Commercial human spaceflight has expanded markedly since 2021, with a growing cohort of civilian participants having undertaken suborbital, orbital free-flyer, and International Space Station visiting-crew missions. Pre-flight medical assessment of this population, however, continues to rely on regulatory guidance that predates the first civilian orbital flight by approximately fifteen years and stratifies eligibility on a single dimension of G-load exposure. The evidence accumulated since on spaceflight-associated neuro-ocular syndrome, on centrifuge-analog tolerance in participants with chronic comorbidities, on the integrated multi-omic findings of recent civilian orbital missions, and on the new mission profiles introduced by the Axiom and Polaris Dawn programs has not been incorporated into a unified evaluation framework. In this narrative review, the aerospace-medicine and clinical literature of the past two decades is synthesized within a framework that distinguishes mission-related background factors (including acceleration, microgravity, spaceflight-associated neuro-ocular syndrome, ionizing radiation, and cabin environment) from participant-related factors (comprising age, sex, reproductive status, and cardiopulmonary, metabolic, and oncological comorbidity). The documented cohort spans 18 to 90 years of age, with the extremes confined to suborbital flights. The orbital free-flyer subset (n=12 across Inspiration4, Polaris Dawn, and Fram2) ranges from approximately 29 to 63 years, and the International Space Station (ISS) visiting-crew subset (n=12 civilian participants across Axiom AX-1 through AX-4, excluding career-astronaut commanders) from 33 to 71 years. Documented comorbidities include treated coronary artery disease, paroxysmal atrial fibrillation, type 2 diabetes mellitus, obstructive sleep apnea, and prior oncological conditions. Based on the available evidence, a clinical routine is proposed, organized across three assessment tiers - a universal baseline (Tier 1), an indication-driven system-specific evaluation (Tier 2), and a mission-class-specific evaluation (Tier 3) - and distributed along a four-step pathway from 90 days before flight to the day of launch. The proposed workflow may serve as an operational starting point, although prospective validation will be required as the commercial era generates additional data on participants, mission profiles, and clinical outcomes.

## Introduction and background

For six decades, human spaceflight was conducted exclusively by government agencies and through a small number of career astronauts selected by multi-year medical and psychological evaluation [1, 2]. This pattern, however, has been recently altered by a rapid succession of commercial missions carrying paying civilian participants into space. The trajectory opened in July 2021, when Blue Origin New Shepard (Blue Origin Enterprises, Kent, USA) performed the first crewed suborbital flight across the Kármán line with civilian passengers aboard, and continued two months later with the SpaceX Inspiration4 mission (Space Exploration Technologies Corporation, Starbase, USA), which completed the first all-civilian orbital flight of the commercial era [3]. By mid-2025, approximately 120 civilian participants had undertaken commercial spaceflight across suborbital, orbital free-flyer, and International Space Station (ISS) visiting-crew missions, a figure that industry and regulatory projections indicate will rise by an order of magnitude within the coming decade [4, 5].

This rapid expansion has introduced a participant population whose clinical profile has not been formally characterized in the aerospace-medicine literature, and whose characteristics differ from those of the career astronaut in every dimension that medical standards have historically used to define eligibility. While the existing evidence base derives almost entirely from career-astronaut cohorts that underwent multi-year medical selection, were aged between thirty and fifty at first flight, and had no clinically manifest disease [1], civilian participants in the commercial era have ranged from eighteen to ninety years of age, with the extremes reached on suborbital flights and the orbital free-flyer and ISS visiting-crew subsets falling within narrower windows of approximately twenty-nine to sixty-three years and thirty-three to seventy-one years, respectively; their documented comorbidities include coronary artery disease, diabetes mellitus, atrial fibrillation, obstructive sleep apnea, and prior oncological conditions [6-8]. The mission profiles undertaken by this heterogeneous population are themselves diverse, comprising suborbital flights of approximately ten minutes in total duration (three to five minutes of which are in microgravity), orbital free-flyer missions of three to five days, and ISS visiting-crew missions of ten to twenty days [3, 9]. Because each profile imposes a distinct physiological load, and because the evidence available for the career-astronaut cohort cannot be extrapolated to a population with markedly different baseline characteristics, the application of career-astronaut medical standards to the commercial spaceflight participant requires explicit reconsideration [4, 10].

The foundational aerospace-medicine consensus for this population is provided by the 2006 US Federal Aviation Administration (FAA) Office of Aerospace Medicine guidance on medical screening of commercial aerospace passengers (DOT/FAA/AM-06/1), which introduced a two-tier approach stratified by G-load exposure (suborbital flights with ≤ +3 Gz, and orbital or high-G flights) and outlined medical history, physical examination, and laboratory components for each tier [11]. Nearly two decades later, however, the FAA document precedes the commercial era inaugurated by the first civilian orbital flight in 2021, and it does not incorporate the subsequent evidence on the systematic characterization of spaceflight-associated neuro-ocular syndrome, on centrifuge-analog tolerance of participants with chronic comorbidities, on the integrated multi-omic findings of the Inspiration4 Space Omics and Medical Atlas [3], or on the mission profiles introduced by the Axiom and Polaris Dawn programs. A structured pre-flight medical evaluation routine that reflects this post-2006 evidence base and the current diversity of mission profiles is therefore not available to clinicians and commercial operators in a consistent format. To address this gap, here we develop, through a narrative review of the literature published between 2006 and 2026 and organized around a clinical framework that distinguishes mission-related background factors from participant-related factors, a synthesis of the evidence that is then operationalized as a tier-based evaluation pathway integrating universal baseline investigations, indication-driven system-specific investigations, and mission-class-specific investigations into a single clinical routine [2, 12].

## Review

Methodology

The literature search underlying the proposed evaluation routine was conducted in accordance with the Scale for the Assessment of Narrative Review Articles (SANRA) reporting framework [13], on the PubMed/MEDLINE database for publications indexed between 1 January 2006 and 31 March 2026, with selected pre-2006 references retained where they remain part of current aerospace-medicine practice. Search terms comprised combinations of "space tourism", "commercial spaceflight", "suborbital flight", "aerospace medicine", "space motion sickness", "spaceflight-associated neuro-ocular syndrome", "centrifuge", "hypergravity", "microgravity AND human", "space radiation AND health", and "fitness to fly AND space", each combined through Boolean operators with condition-specific modifiers covering cardiovascular disease, diabetes mellitus, obstructive sleep apnea, pregnancy, and cancer survivorship. The Cochrane Central Register of Controlled Trials was queried in parallel for interventional data on in-flight medical countermeasures, and the NASA Technical Reports Server was queried for operational and physiological reports from career-astronaut programs not indexed in PubMed/MEDLINE. Grey literature was retrieved from the Aerospace Medical Association position statements, the NASA Space Flight Human-System Standard (NASA-STD-3001), Federal Aviation Administration guidance for commercial human spaceflight (including the 2006 DOT/FAA/AM-06/1 document that constitutes the foundational regulatory consensus for this population) [11], and peer-reviewed technical reports of the National Aerospace Training and Research Center; aerospace medicine guidance from the European Space Agency, the Japan Aerospace Exploration Agency, the Russian Institute of Biomedical Problems, and the International Academy of Aviation and Space Medicine was not systematically covered by this retrieval. Eligible sources were original research, systematic and narrative reviews, consensus statements, and case reports published in English-language peer-reviewed venues, together with the grey literature documents listed above. Excluded were animal-only studies lacking translational framing, non-peer-reviewed abstracts, and conference proceedings without full-text availability. When competing estimates were identified, in particular for Apollo-era cardiovascular mortality and for the dose-duration relationship of spaceflight-associated neuro-ocular syndrome, both sources were retained, and their methodological limitations were addressed in the relevant sections of the review.

Professional versus commercial spaceflight

Table 1 summarizes the principal clinical differences between the career astronaut and the commercial spaceflight participant across eight dimensions relevant to medical selection and evaluation.

**Table 1:** Principal clinical differences between the career astronaut and the commercial spaceflight participant across eight dimensions relevant to pre-flight medical evaluation. *Dimensions marked with an asterisk denote the three axes of primary operational importance that motivate the development of a distinct evaluation routine for the commercial spaceflight participant, as discussed in the main text. Abbreviations: HbA1c, glycated hemoglobin; ISS, International Space Station.

#	Dimension	Career astronaut	Commercial spaceflight participant
1	Selection pathway*	Multi-year medical and psychological assessment conducted by the space agency	Pre-flight evaluation compressed into weeks to months, coordinated by the commercial operator and the evaluating physician
2	Baseline health status*	Categorical exclusion of clinically manifest disease; "medically normal"	Retention of well-controlled comorbidities on a case-by-case basis; "medically controlled"
3	Decision logic	Exclusion-based eligibility	Risk-stratified eligibility with mission-class-specific calibration
4	Age at first flight	Typically 30 to 50 years	Documented range 18 to 90 years; mean approximately 50 to 55 years
5	Sex distribution	Historically fewer than 15% women	Approximately 25% women, with increasing representation in recent orbital missions
6	Acceptable comorbidity burden	None at the time of selection	Treated hypertension, stable coronary disease after revascularization, paroxysmal atrial fibrillation on rate or rhythm control, stable valvular disease, type 2 diabetes with HbA1c < 7.5%, hypothyroidism on stable replacement, obstructive sleep apnea on therapy, prior treated oncological disease
7	Mission duration*	Months in low Earth orbit; years projected for deep-space missions	Approximately 3 to 5 minutes of microgravity in suborbital flight, 3 to 5 days in orbital free-flyer missions, 10 to 20 days in ISS visiting-crew missions
8	Post-flight surveillance	Longitudinal occupational health follow-up over decades	Time-limited post-flight assessment, typically over weeks to months, with no standardized long-term registry to date

Three axes are of primary operational importance and motivate the need for a distinct evaluation routine. The first is the depth and duration of medical screening, which for the career astronaut comprises a multi-year assessment with categorical exclusion of clinically manifest disease, whereas for the commercial participant, the screening interval is compressed into weeks or months and allows retention of well-controlled comorbidities on a case-by-case basis [1, 8, 14]. The second is the baseline health status, which for the career astronaut approximates absolute absence of disease, whereas the commercial participant is frequently medically controlled rather than medically normal, with consequent shift from exclusion-based to risk-stratified decision-making [15, 16]. The third is the mission duration, which varies by up to three orders of magnitude across the commercial spectrum (five minutes of microgravity in suborbital flight, days to weeks in orbital and ISS visiting-crew missions) and calls for a mission-class-specific evaluation component that the uniform career-astronaut standards do not provide [3, 17]. These three axes together define an operational profile that cannot be addressed by a simple relaxation of the career-astronaut standards, because each axis requires a different type of adaptation: the first calls for a compressed but structured sequence of investigations, the second for an evidence-based articulation of risk stratification across organ systems, and the third for a mission-class-specific component layered onto the preceding two. A pre-flight medical evaluation routine tailored to the commercial spaceflight participant must therefore combine these three adaptations within a single coherent workflow, rather than preserve the categorical eligibility logic of the career-astronaut standards in attenuated form.

Review framework

In this review, we distinguish two groups of factors relevant to the evaluation of the commercial spaceflight participant. The first group comprises the mission-related background factors, which include acceleration loading during launch and re-entry, microgravity, spaceflight-associated neuro-ocular syndrome, ionizing radiation, and cabin environmental conditions, and whose magnitude scales predominantly with the mission profile rather than with the individual participant. The second group comprises the participant-related factors, which include age, sex, reproductive status, cardiopulmonary and metabolic comorbidity, and prior oncological or hematological conditions, and whose relevance is largely independent of the mission class. This distinction extends the 2006 FAA guidance on medical screening of commercial aerospace passengers, which stratified eligibility on a single variable of G-load exposure, by introducing the participant group as a co-determinant of risk and by subdividing the mission group into five physiologically distinct exposure domains rather than a binary G-load cut-off [11]. A mission-class decision and a comorbidity decision can thus be taken separately within this structure, even when they apply to the same candidate, and combined only at the level of the final evaluation output. The framework does not attempt to weight the two groups of factors against each other numerically, because the available evidence is not uniform enough to support a quantitative risk model, and a semi-quantitative assignment of risk within each group is the highest resolution currently achievable. The framework is operationalized as a three-tier pre-flight evaluation pathway, presented in the Pre-Flight Medical Evaluation section.

Mission-related factors

Launch and Re-entry: Acceleration and G-Induced Loss of Consciousness

Acceleration is the first stress of a spaceflight that the candidate physically perceives, and historically, it has been the principal motive for excluding individuals with cardiovascular limitations from astronaut selection. It is also the only one of the spaceflight stressors whose tolerance can be measured directly in the individual before flight, through centrifuge exposure, and whose magnitude varies markedly between commercial vehicles currently in service. The Virgin Galactic rocket-plane produces a combined profile of head-to-foot (+Gz) and chest-to-back (+Gx) acceleration, with peak values in the order of +4 Gz at engine ignition and +6 Gx during atmospheric re-entry, whereas the Blue Origin New Shepard capsule produces a predominantly +Gx profile with peak values in the order of +3 to +4 Gx during both ascent and descent [18]. Acceleration tolerance in healthy individuals is conservatively approximated at +3 Gz for rapid-onset profiles and +3.5 Gz for gradual-onset profiles, with tolerance shifting upward by approximately one G during +Gx loading because the vascular column between the heart and the brain is not aligned with the acceleration vector [19]. The most informative human evidence on commercial-analog exposure comes from centrifuge simulation at the National Aerospace Training and Research Center, where consecutive cohorts of medically screened volunteers, including participants with well-controlled cardiovascular, pulmonary, and endocrine comorbidities, completed suborbital and orbital acceleration profiles (Table 2) [15, 18].

**Table 2:** Centrifuge cohort studies of relevance to the commercial spaceflight participant. All available evidence derives from self-selected volunteers at two centrifuge facilities and provides an upper bound on tolerability rather than a population-wide estimate. Abbreviations: +Gx, chest-to-back acceleration; +Gz, head-to-foot acceleration; NASTAR, National AeroSpace Training and Research Center; QinetiQ, United Kingdom Defence Evaluation and Research Agency successor entity.

Study	Year	Facility	Cohort size	Profile tested	Population characteristics	Principal finding
Blue et al.	2012	NASTAR (United States)	Approximately 80	Combined +Gz and +Gx, Virgin Galactic suborbital analog	Medically screened civilian volunteers	Validation of acute tolerability for the Virgin Galactic suborbital acceleration profile in untrained civilian participants
Blue et al.	2014	NASTAR (United States)	86	Suborbital and orbital +Gz and +Gx profiles	Medically screened, including well-controlled cardiovascular, pulmonary, and endocrine comorbidities	Acceptable acute tolerability across the cohort, including in participants with controlled comorbidity burden
Blue et al.	2017	NASTAR (United States)	Approximately 50	Suborbital training and familiarization profile	NASTAR cohort follow-up, naive participants	Pre-flight training reduced anxiety and improved task performance
Smith et al.	2022	QinetiQ (United Kingdom)	24	Suborbital +Gz with combined hypoxia at 15% inspired oxygen	Healthy volunteers under simulated cabin altitude	Single documented episode of G-induced loss of consciousness; non-sustained supraventricular dysrhythmias in a minority of runs, resolved on deceleration

A single documented episode of G-induced loss of consciousness was recorded in 24 participants under combined hypoxia at 15% inspired oxygen, and non-sustained supraventricular dysrhythmias were observed in a minority of runs and resolved on deceleration [17, 20]. These centrifuge data derive from fewer than two hundred self-selected volunteers at a single facility and therefore establish an upper bound on tolerability rather than a population-wide estimate, and the low event rate should be interpreted as consistent with the absence of unexpected intolerance in a screened cohort rather than as a guarantee of safety at a larger scale. Countermeasures applicable to the commercial setting include anti-G straining maneuvers adapted to untrained individuals, among which a simplified sequence combining lower-limb muscle contraction with slow rhythmic breathing has been shown to raise relaxed tolerance by approximately one G in naive subjects [21]. Pre-flight training and familiarization further reduce anxiety and improve task performance in naive participants during centrifuge simulation, supporting their inclusion as preparatory countermeasures alongside the physiological maneuvers [14, 22]. The available evidence, therefore, indicates that the risk of acceleration-related adverse events during commercial suborbital flight is low in a medically screened population, but not uniform across vehicle configurations. Pre-flight estimation of that risk requires a combined appraisal of the acceleration profile of the chosen vehicle and of the individual circulatory reserve of the candidate.

Microgravity: Cardiovascular and Fluid-Volume Adaptation

The exposure to microgravity is what most distinctively defines a spaceflight as such, and its clinical relevance for the commercial setting depends almost entirely on the duration of the mission, which in current commercial profiles spans three orders of magnitude from approximately five minutes during a suborbital flight to two weeks or more during an International Space Station visit. Pre-flight evaluation of the candidate's cardiovascular and fluid-volume reserve is therefore weighted by mission class rather than applied uniformly, and the questions that the evaluating physician asks of an Inspiration4-type free-flyer participant are not the same as those asked of an ISS visiting-crew candidate. The acute physiological response to microgravity is dominated by a cephalad redistribution of approximately one to two liters of intravascular and interstitial fluid from the lower body to the thorax and head, with consequent reduction in plasma volume of about 10% to 15% over the first 24 to 72 hours, mediated by the diuretic effect of central volume receptor activation [10]. In short-duration missions of three to five days, the fluid shift remains functionally compensated in healthy individuals, and the principal post-flight finding is a transient orthostatic intolerance on return that resolves within hours to days [9]. The integrated multi-omic and physiological data from the Inspiration4 mission (n=4 crew members) confirmed that a three-day orbital flight produces measurable cardiovascular, fluid, and immune changes in untrained civilian participants, but that the majority of these changes returned to baseline within months and none required clinical intervention [3]. Mission durations exceeding two weeks introduce additional cardiovascular deconditioning, including reductions in left-ventricular mass and aerobic capacity that have been documented in career astronauts after ISS expeditions and that are clinically relevant for the visiting-crew profile of commercial flights [1, 14]. The available evidence indicates that the cardiovascular and fluid-volume effects of microgravity are well tolerated in screened individuals during short orbital missions. Pre-flight evaluation, therefore, focuses on candidates whose baseline volume regulation or orthostatic control is impaired, and the depth of post-flight monitoring is calibrated to the duration of the mission rather than applied uniformly.

Spaceflight-Associated Neuro-Ocular Syndrome

Sustained cephalad fluid redistribution under microgravity is thought to raise intracranial pressure and the pressure gradient across the optic nerve head, and the ophthalmic signs that follow have been grouped under the label spaceflight-associated neuro-ocular syndrome [23]. The constellation includes optic disc edema, posterior globe flattening, choroidal and retinal folds, hyperopic refractive shifts, cotton-wool spots, and distention of the optic nerve sheath, and it has been systematically described in long-duration career astronauts after missions of four months or longer on the International Space Station [23, 24]. The incidence of one or more of these findings in that population approaches 40% to 60%, with a clear but incompletely quantified relationship between cumulative exposure time in microgravity and the severity of the imaging findings, and with partial but not universal reversibility over months to years after return [1, 10, 24]. The pathophysiological mechanisms connect spaceflight ophthalmic findings with chronic intracranial hypertension and with terrestrial ocular conditions characterized by elevated translaminar pressure differences [25]. Predictive models developed in career-astronaut cohorts have identified pre-flight intraocular pressure, baseline cup-to-disc ratio, and sex as candidate risk modifiers, although the small sample sizes of available cohorts limit the precision of these estimates [26]. Evidence in short-duration civilian flight is limited to the four participants of the Inspiration4 mission, in whom no clinically relevant ophthalmic changes were documented during the three-day orbital exposure [3]. This finding is consistent with the duration-dependent pattern observed in career astronauts, but it does not support a confident extrapolation to the absence of risk, because the sample size is small and the post-flight follow-up window was not designed to capture delayed findings. Pre-flight ophthalmological assessment with optical coherence tomography, visual acuity, and fundoscopy establishes a baseline against which any post-flight change can be referenced, and the depth of post-flight monitoring is calibrated to the duration of the mission. The available evidence indicates that spaceflight-associated neuro-ocular syndrome is unlikely to develop clinically during suborbital or short orbital flight, and that it becomes a material consideration in planning for International Space Station visiting-crew missions of two weeks or longer. Post-flight follow-up at three and six months is therefore offered to participants of longer orbital and visiting-crew missions, and baseline optical coherence tomography is integrated into the indication-driven component of the evaluation for every candidate with documented ophthalmic risk factors.

Radiation: Dose Stratification by Mission Class

Unlike acceleration and microgravity, both of which produce acute physiological responses with measurable individual tolerance thresholds, ionizing radiation acts on the commercial spaceflight participant as a cumulative exposure with no clinically perceptible acute manifestations, and its consequences are statistical rather than mechanistic. Pre-flight evaluation, therefore, turns less on screening for low individual tolerance and more on documenting the small set of conditions, principally pregnancy and prior therapeutic radiation exposure, that meaningfully modify the cancer and reproductive risk associated with the cumulative dose of the chosen mission. The radiation environment of low Earth orbit is dominated by galactic cosmic radiation and by trapped protons in the South Atlantic Anomaly, with episodic contributions from solar particle events that can produce dose rates several orders of magnitude above the background within hours [10]. Suborbital flights of approximately ten minutes contribute a negligible additional dose of less than 0.1 mSv per flight, which is comparable to a single transcontinental commercial airline flight [4]. Orbital free-flyer missions of three to five days have been associated with cumulative doses in the range of 0.7 to 1.5 mSv, as documented during the Inspiration4 mission (n=4 crew members), with the spread reflecting orbital altitude, inclination, and the duration of passage through the South Atlantic Anomaly [3]. International Space Station visiting-crew missions of 10 to 20 days deliver cumulative doses of approximately 3 to 8 mSv, which remain well below the 50 mSv annual occupational limit applied to career astronauts but exceed the 1 mSv annual limit applied to the general public under standard radiological protection criteria [5, 14]. The long-term oncological and cardiovascular consequences of these acute exposures in civilian populations are not directly known, and the small Apollo cohort in which an excess of cardiovascular mortality has been reported (n = 7) is too limited to support causal inference about radiation-mediated vascular injury [27]. The available evidence indicates that the cumulative radiation dose of a single commercial spaceflight is low in absolute terms and well below career-astronaut occupational limits, but is not negligible for pregnancy or for individuals with documented prior therapeutic radiation exposure. Pre-flight evaluation, therefore, includes documentation of pregnancy status and of prior radiotherapy, and the candidate is informed of the cumulative dose expected for the chosen mission class.

Cabin Environment: Atmospheric Composition and Pressure Integrity

The cabin environment differs from the other three background domains in that the risk it carries is not an intrinsic property of spaceflight but a consequence of the vehicle's life-support system, and it becomes clinically relevant only when that system operates at its design limits or fails. Pre-flight evaluation, therefore, intersects this domain at two narrow points, namely the tolerance of the candidate to the elevated carbon-dioxide concentrations that are typical of long-duration orbital environments, and the capacity to respond to a rapid decompression event should vehicle pressure integrity be lost. Ambient carbon dioxide on board the International Space Station routinely ranges between 2500 and 5000 parts per million, approximately ten times the terrestrial baseline, and has been associated with headache, reduced cognitive throughput, and mild visual disturbance in a proportion of long-duration crewmembers [28]. Whether these effects extend to the 10- to 20-day visiting-crew profile typical of commercial ISS missions has not been directly measured, and current recommendations are extrapolated from longer-duration data. The risk of sudden cabin depressurization, although low in modern vehicles, cannot be eliminated, and pre-flight familiarization with the pressure suit or oxygen mask of the chosen vehicle is part of the mission-class-specific component of the evaluation [4]. The available evidence indicates that the cabin environment does not call for systematic pre-flight testing of the candidate's tolerance, but mission-class counseling on the expected carbon-dioxide profile and on the decompression protocols of the vehicle is part of a complete evaluation.

Participant-related factors

Demographic Profile

The age distribution of the commercial spaceflight participant population extends across a range that has no precedent in the career astronaut literature. Documented participants in suborbital and orbital commercial missions have spanned from 18 years (Oliver Daemen, the youngest civilian to cross the Kármán line aboard Blue Origin in July 2021) to 90 years (William Shatner, the oldest individual to complete a suborbital flight, on Blue Origin in October 2021) [4, 8]. The mean age of the commercial spaceflight cohort has been estimated at approximately 50 to 55 years, roughly a decade above the mean age at first flight of the career astronaut, with a pronounced right skew that reflects the concentration of paying participants in older and wealthier segments of the general population [6, 8]. The sex distribution has remained unbalanced in favor of male participants in a ratio of approximately three to one, although the Inspiration4, Polaris Dawn, and Fram2 missions have contributed an increasing proportion of women participants, including the first commercial extravehicular activity performed by a woman during the Polaris Dawn mission [3]. The civilian orbital free-flyer cohort has reached twelve participants across Inspiration4 (n=4), Polaris Dawn (n=4), and Fram2 (n=4), and the parallel ISS visiting-crew programme has added four Axiom Space missions of approximately ten days each (Table 3).

**Table 3:** Commercial spaceflight missions, July 2021 to mid-2025, identified in the present review. Aggregate civilian spaceflight participant count across the period is approximately 120, of whom approximately 25 completed orbital free-flyer or ISS visiting-crew profiles. Abbreviations: ISS, International Space Station; NASA, National Aeronautics and Space Administration.

Mission	Operator	Launch date	Class	Duration	Civilian crew (n)	Notes
Inspiration4	SpaceX	15 Sep 2021	Orbital free-flyer	3 days	4	First all-civilian orbital mission
AX-1	Axiom Space / SpaceX	8 Apr 2022	ISS visiting-crew	17 days	4 (incl. retired-NASA commander)	First commercial ISS visiting-crew mission
AX-2	Axiom Space / SpaceX	21 May 2023	ISS visiting-crew	9 days	4 (incl. retired-NASA commander)	Routine commercial mission
AX-3	Axiom Space / SpaceX	18 Jan 2024	ISS visiting-crew	18 days	4 (incl. retired-NASA commander)	Routine commercial mission
Polaris Dawn	SpaceX	10 Sep 2024	Orbital free-flyer	5 days	4	First commercial extravehicular activity
Fram2	SpaceX	31 Mar 2025	Orbital free-flyer (polar)	4 days	4	First crewed polar-retrograde orbit
AX-4	Axiom Space / SpaceX	25 Jun 2025	ISS visiting-crew	~14 days	4 (incl. retired-NASA commander)	Routine commercial mission
Suborbital flights (aggregate)	Virgin Galactic (VSS Unity, Galactic 01–07) + Blue Origin (New Shepard NS-16 to NS-32)	Jul 2021 – Jun 2025	Suborbital	~5 to 10 minutes of microgravity	Approximately 95 across approximately 20 crewed flights	Includes the 18-year-old (Oliver Daemen, NS-16) and 90-year-old (William Shatner, NS-18) extremes of the documented age range

A proof-of-concept study from the AX-1 mission documented musculoskeletal pain and short-term sensory alterations in two participants during and after a 17-day flight, with return to baseline by three months [29]. Integrated data from the orbital free-flyer programme have begun to characterize the short-duration spaceflight phenotype on multiple physiological axes. High-resolution peripheral quantitative computed tomography in the eight Polaris Dawn and Fram2 crew members (four men, four women) documented significant tibial bone density and microarchitectural changes following 3 to 5 days of microgravity, while the distal radius remained unchanged, indicating early onset of weight-bearing skeletal deterioration even after brief exposure [30]. Cardiopulmonary resuscitation feasibility testing during Polaris Dawn established the outboard seat of the Dragon spacecraft as the ergonomically preferred site for chest compressions, with adequate compression frequency achieved by all four crew members but with documented elevation of cabin carbon dioxide and vehicle accelerations attributable to the task [31]. Pharmaceutical content analysis of nineteen medications subjected to vacuum exposure during the Polaris Dawn extravehicular activity showed no clinically meaningful degradation, supporting the feasibility of repackaged medical kits for commercial and exploratory missions [32]. Unlike the career astronaut population, in which the absence of clinically manifest disease has been a condition of selection, commercial spaceflight participants have included individuals with previously treated breast cancer, chronic hypertension on pharmacological therapy, and a history of coronary revascularization, all of whom completed their missions without documented acute medical events [6, 8, 15]. The available evidence, therefore, indicates that the commercial spaceflight participant population is appreciably older, more medically heterogeneous, and more demographically skewed than the career astronaut cohort on which current aerospace-medicine standards are based. A pre-flight evaluation routine for this population must accommodate that heterogeneity without reverting to categorical exclusion, because the empirical record shows that medically controlled conditions are compatible with flight under mission-class-appropriate screening.

Cardiopulmonary Comorbidities

Cardiopulmonary conditions represent the prevalent comorbidity class within the commercial spaceflight participant population documented to date, and their distribution across recent cohorts establishes the empirical starting point for risk-stratified pre-flight evaluation. Participants with treated hypertension, chronic stable coronary artery disease after complete revascularization, paroxysmal atrial fibrillation controlled on rate or rhythm strategies, and clinically stable valvular heart disease have completed suborbital and short-duration orbital missions without documented acute cardiovascular events [6, 8, 15]. Centrifuge simulation cohorts at the National Aerospace Training and Research Center have included participants with hypertension, prior coronary revascularization, and prior cardiac surgery, and have recorded a low rate of non-sustained supraventricular dysrhythmias during high-G profiles, together with a single documented G-induced loss of consciousness under combined hypoxia at 15% inspired oxygen [15, 17, 18, 20]. These cardiovascular data derive from self-selected volunteers at a single facility and should be read as evidence of acceptable acute tolerability in a screened cohort rather than as a population-wide safety estimate, because the absence of recorded events in fewer than two hundred participants does not rule out rare but clinically important adverse outcomes. Pulmonary comorbidities relevant to commercial spaceflight include obstructive sleep apnea, stable asthma, mild to moderate chronic obstructive pulmonary disease, and restrictive patterns of varied etiology, each of which can be characterized preflight by spirometry and by the hypoxic altitude simulation test modeled on an inspired oxygen fraction of approximately 15.1%, which corresponds to the typical cabin altitude of 8000 feet used in orbital and suborbital vehicles [4, 10]. A post-test arterial oxygen saturation below 92% to 95% or a forced expiratory volume in one second below 50% of predicted identifies candidates who require either supplemental oxygen planning or exclusion from flight, depending on the mission profile and the reversibility of the underlying condition [9]. The available evidence indicates that medically controlled cardiopulmonary disease is compatible with commercial suborbital and short orbital flight in a screening context that applies indication-driven investigations, rather than categorical exclusion based on the diagnostic label alone. Decompensated heart failure, unstable coronary syndrome, severe pulmonary hypertension, and uncontrolled obstructive airway disease remain clear contraindications, whereas most intermediate phenotypes are resolved through the Tier 2 indication-driven investigations that couple functional reserve testing to the acceleration and altitude profile of the chosen mission.

Metabolic and Endocrine Conditions

The metabolic and endocrine conditions most likely to be encountered in the commercial spaceflight participant population are type 2 diabetes mellitus, primary hypothyroidism, and, to a lesser extent, primary adrenal insufficiency, all of which reach a cumulative prevalence of approximately 15 to 25% in the fifth and sixth decades that correspond to the mean age of current commercial participants [8, 15]. Stable type 2 diabetes with glycated hemoglobin below 7.5% and no history of severe hypoglycemia is compatible with suborbital and short-duration orbital flight, whereas insulin-dependent regimens require a mission-class-specific evaluation that addresses continuous glucose monitoring under microgravity, pump infusion-site stability during launch acceleration, and counter-regulatory reserve during post-flight orthostatic adaptation [4]. Hypothyroidism on stable levothyroxine replacement with normal thyrotropin does not restrict eligibility, whereas inadequately controlled hyperthyroidism increases the risk of supraventricular arrhythmia during acceleration and is treated as a temporary contraindication until euthyroid status is reestablished [10]. Both primary adrenal insufficiency and long-term pharmacological glucocorticoid dependence modify the acute stress response to spaceflight, and require a documented stress-dose protocol for launch day and for any in-flight medical event, irrespective of mission duration [6]. The available evidence indicates that most metabolic and endocrine conditions encountered in the commercial population are compatible with flight when biochemical control is documented, and the relevant counter-regulatory response is preserved. Indication-driven investigations directed at glycemic stability, thyroid function, and adrenal reserve, therefore, replace the blanket exclusion that has been applied to endocrine disease in the career astronaut setting, and align the evaluation pathway with the phenotype rather than with the diagnostic label.

Age, Sex, and Reproduction

Age and sex exert effects on aerospace tolerance that are, in most cases, continuous rather than categorical, and their interpretation for the commercial spaceflight participant has to contend with an evidence base in which women and individuals above the sixth decade are markedly under-represented. Advancing age is associated with a progressive decline in orthostatic tolerance, in maximal aerobic capacity, and in skeletal mineral density, together with a higher cumulative prevalence of the cardiopulmonary and metabolic conditions relevant to pre-flight evaluation, all of which justify a lower threshold for indication-driven investigations rather than an age cut-off [1, 8]. Empirical flight experience does not support a chronological age limit per se, as documented participants have included individuals at both extremes of the range without recorded acute intolerance, and the operational question is therefore the physiological age of the candidate rather than the calendar age [4]. Sex-related differences relevant to spaceflight include a roughly two-fold higher incidence of post-bed-rest orthostatic intolerance in women, a more rapid rate of spaceflight-related bone loss at weight-bearing sites, and an evidence base on spaceflight-associated neuro-ocular syndrome that derives almost entirely from male career astronauts and that cannot be extrapolated to women without caveat [14, 33]. The systematic application of sex- and gender-based analytical methodology to aerospace research provides the framework for characterizing these differences as the cohort of women participants expands [34].

Reproductive status introduces a categorical constraint that the other participant factors do not impose. Pregnancy at any gestational stage is an absolute contraindication to spaceflight, because the cumulative radiation dose to the conceptus during a single low-Earth-orbit mission can exceed the 1 mSv occupational limit recommended for pregnancy by international radiological protection guidelines [35], a ceiling whose practical application has been demonstrated in commercial aviation for pregnant aircrew and is correspondingly more stringent in the higher-dose spaceflight environment [36]. Contraception counseling is therefore integral to the evaluation of women of reproductive age who are scheduled for flight within 12 months. The integrated Inspiration4, Polaris Dawn, and Fram2 datasets have begun to close the sex-specific evidence gap for short-duration orbital missions, but the sample size per sex stratum remains small, and the aggregate conclusion is that current tolerability estimates in women are consistent with those in men at the level of the group, while individual variability is less characterized. The available evidence indicates that chronological age and sex are not eligibility criteria in themselves, but both guide the depth and the specific targets of the indication-driven and mission-class-specific components of the evaluation. Pregnancy testing and contraception counseling remain required elements of the universal baseline for every woman of reproductive age who is evaluated for a commercial spaceflight.

Additional Clinical Subgroups

Several additional phenotypes can be encountered in the commercial spaceflight participant population with a frequency that requires explicit handling within the pre-flight evaluation. Bleeding disorders and long-term anticoagulation warrant pre-flight assessment of the trauma risk associated with acceleration loading and of the reversibility of the anticoagulant regimen in case of in-flight emergency. Cancer survivorship involves documentation of prior chemotherapy and radiotherapy exposure, surveillance status, and functional recovery, rather than a categorical exclusion by diagnosis [6, 8]. A history of seizures, transient ischemic attack, stroke, or severe migraine requires specialist neurological review and a stability interval appropriate to the mission class, and psychiatric conditions with a plausible trigger in confinement or sensory overload require documented stability on current therapy before eligibility is confirmed [4]. All these subgroups enter the evaluation through the Tier 2 indication-driven pathway, with the specific investigations dictated by the phenotype rather than by the label.

Spaceflight-associated clinical considerations

Space Motion Sickness

Space motion sickness has the highest reported incidence of any clinical syndrome of early spaceflight, affecting between 60% and 80% of crewmembers within the first 72 hours of microgravity, and it is at the same time the syndrome with the least restrictive implications for pre-flight eligibility because of its self-limited course and the availability of effective pharmacological countermeasures [1, 10]. The mechanism is neurovestibular conflict between otolith and visual inputs under altered gravitational reference, with symptoms that include nausea, pallor, cold sweating, and vomiting, and that typically resolve over 48 to 72 hours as central recalibration occurs [6]. Pre-flight susceptibility can be estimated with the Motion Sickness Susceptibility Questionnaire, which has been validated against zero-G parabolic flight exposure in 246 participants and which identifies higher-susceptibility candidates while also demonstrating the protective effect of older age and prior zero-G experience [37, 38]. In the same parabolic cohort, scopolamine prophylaxis reduced mean sickness rating from 2.93 to 1.81 on a six-point scale and lowered the incidence of vomiting, supporting its routine pre-flight offer for commercial suborbital and short orbital profiles [37]. For suborbital flights of approximately ten minutes, symptoms appear mainly on egress from microgravity rather than during the short in-flight exposure, whereas for orbital and ISS visiting-crew missions, adaptation takes place in flight, and post-flight residual symptoms are uncommon [4]. The available evidence indicates that space motion sickness does not restrict eligibility for commercial spaceflight in an otherwise fit candidate, and that pre-flight assessment is limited to documented screening with the Motion Sickness Susceptibility Questionnaire and counseling on scopolamine or equivalent transdermal prophylaxis. The occurrence of severe symptoms in a minority of participants is the expected norm rather than a sign of failed selection.

Immune Dysregulation

The evidence base on spaceflight-associated immune dysregulation derives almost entirely from International Space Station expeditions of six months or longer, and its direct applicability to commercial spaceflight of days to two weeks is therefore limited [1]. The integrated multi-omic dataset from the Inspiration4 mission (n=4 crew members) documented measurable changes in cytokine profiles and leukocyte subsets during a three-day orbital flight, but these changes returned to baseline within weeks and did not translate into clinically relevant infectious or inflammatory events [3]. Pre-flight evaluation for the commercial spaceflight participant, therefore, concerns two practical points, namely the review of routine adult vaccination status in line with standard travel-medicine practice, and the identification of any active infectious condition that would warrant a temporary contraindication until resolution [4, 10]. The available evidence does not support additional immunological testing or prophylactic antiviral coverage in the screened candidate for short-duration commercial missions.

Pharmacology and Drug Considerations

Pharmacological considerations in commercial spaceflight differ from the corresponding literature in career astronaut medicine, which has concentrated on in-flight countermeasures for a pre-selected healthy cohort. For the commercial participant, who frequently arrives for evaluation on chronic therapy, the operational priority is the continuity and appropriate handling of ongoing medications rather than the introduction of novel prophylactic agents. The review of current medications is therefore a component of the universal baseline, and covers dose, timing, mode of administration, and the interval since the last change of regimen [4, 7]. Two classes of pharmacokinetic concern apply during the acute phase of spaceflight, namely the altered volume of distribution for hydrophilic drugs caused by cephalad fluid redistribution, and the reduction or delay in oral absorption caused by the gastric motility changes associated with space motion sickness, with potential consequences for narrow therapeutic-index medications such as anticoagulants and antiarrhythmic drugs [10]. Candidates on oral anticoagulation, insulin pumps, continuous glucose monitoring systems, and inhaled therapies require a mission-class-specific continuity plan that addresses in-flight dosing, device tolerance of launch acceleration, and contingency procedures in case of device failure or missed dose [15]. Pre-flight centrifuge familiarization is informative for candidates on cardiovascular therapy with a potential impact on acceleration tolerance, and the threshold for referral should be individualized on the basis of the specific regimen and the mission profile rather than applied as a generic requirement [1, 20]. The available evidence indicates that most established chronic therapies are compatible with commercial spaceflight when continuity is planned, but that the pre-flight evaluation cannot stop at the medical-history form alone.

Pre-flight medical evaluation: a tier-based routine

Structure of the Evaluation Pathway

**Figure 1:**
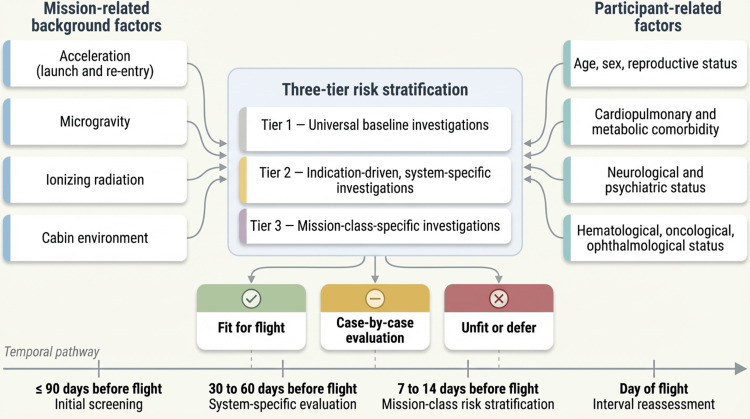
Clinical framework for pre-flight medical evaluation of the commercial spaceflight participant. The figure summarizes the conceptual framework developed in this review and operationalized as a tier-based clinical routine. Two groups of factors enter the evaluation from opposite sides, namely the mission-related background factors (acceleration during launch and re-entry, microgravity, spaceflight-associated neuro-ocular syndrome, ionizing radiation, and cabin environmental conditions), whose magnitude scales with the mission profile rather than with the individual, and the participant-related factors (age, sex, reproductive status, and cardiopulmonary, metabolic, neurological, psychiatric, hematological, oncological, and ophthalmological status), whose relevance is largely independent of the mission class. Both groups converge on a three-tier risk-stratification process, in which Tier 1 comprises the universal baseline investigations applied to every candidate, Tier 2 comprises the indication-driven system-specific investigations triggered by clinical findings at Tier 1 or by pre-existing diagnoses, and Tier 3 comprises the mission-class-specific investigations calibrated to suborbital flight, orbital free-flyer missions, or International Space Station visiting-crew missions. The output of the tier-based process is an eligibility decision expressed in one of three categories, namely fit for flight, case-by-case evaluation, or unfit or defer. The decision is applied across a four-step temporal pathway that begins with initial screening no later than 90 days before flight, continues with system-specific evaluation at 30 to 60 days, is followed by mission-class risk stratification at 7 to 14 days, and is completed by a day-of-flight reassessment that confirms interval stability and documents fitness for launch. Image credits: Piercarlo Minoretti (Created with FigureLabs.ai).

The pre-flight medical evaluation of the commercial spaceflight participant is proposed as a four-step pathway distributed over the months preceding flight. Initial screening is performed no later than 90 days before the scheduled launch window and identifies any condition that requires extended workup or stabilization. System-specific evaluation is completed 30 to 60 days before flight and is triggered by findings from screening or by pre-existing diagnoses. Mission-class risk stratification, including analog exposure where available, is conducted 7 to 14 days before flight. A day-of-flight reassessment is performed to confirm interval stability and to document fitness for launch. The evaluation is organized around three assessment tiers, each building on the previous without replacing it, and the tiers are independent axes, namely universal baseline investigations (Tier 1), investigations triggered by clinical indication (Tier 2), and investigations triggered by mission class (Tier 3) (Figure 1).

Tier 1 Universal Baseline Assessment

Tier 1 investigations are applied to every commercial spaceflight participant, independently of prior medical history or mission class, and establish the physiological baseline against which in-flight and post-flight changes can be interpreted. The core battery comprises a standardized clinical history with a structured questionnaire covering cardiovascular, pulmonary, metabolic, endocrine, neurological, psychiatric, ophthalmic, and reproductive domains, a complete physical examination, and a 12-lead resting electrocardiogram [1, 39]. Imaging includes a chest radiograph to exclude occult pulmonary or mediastinal disease [1]. Pulmonary baseline is established through spirometry, with measurement of forced expiratory volume in one second, forced vital capacity, and their ratio [40, 41]. Laboratory investigations comprise a complete blood count, a comprehensive metabolic panel including electrolytes, hepatic and renal function, glycated hemoglobin, fasting lipid profile, and urinalysis [1]. A quantitative serum or urinary β-human chorionic gonadotropin assay is required for all women of reproductive age, performed within 72 hours of flight, on the grounds of radiation exposure, acceleration tolerance, and the absence of obstetric emergency-care capability [34, 42]. Ophthalmic baseline comprises visual acuity with current refractive correction, tonometry, and dilated funduscopy [23, 24]. Auditory baseline is established with pure-tone audiometry. Neurovestibular susceptibility is documented using the Motion Sickness Susceptibility Questionnaire, recognizing that the instrument does not predict in-flight sickness with sufficient specificity to exclude symptomatic participants and is therefore used for counseling and countermeasure planning rather than for disqualification [37, 43]. Psychological screening is completed with a validated anxiety inventory because psychiatric history and self-reported symptoms do not reliably predict in-flight anxiety, and a positive screen triggers specialist evaluation rather than categorical exclusion [14].

Tier 2 System-Specific Assessment

Tier 2 investigations are triggered by findings from Tier 1 or by a documented medical history, and they are indication-driven rather than mission-class-driven. In the cardiovascular domain, transthoracic echocardiography is indicated when Tier 1 identifies structural or functional abnormalities, or in the presence of a cardiac history; exercise tolerance testing is indicated for candidates with cardiovascular risk factors or with symptoms consistent with ischemia; 24-hour ambulatory electrocardiography is indicated when arrhythmias are documented or suspected; cardiac magnetic resonance is reserved for specific indications such as suspected infiltrative or inflammatory cardiomyopathy [8, 20, 39]. In the pulmonary domain, the hypoxia-altitude simulation test is indicated when forced expiratory volume in one second is below 50% of predicted, or when resting peripheral oxygen saturation on room air is 92% to 95%, or in the presence of established pulmonary disease with preserved spirometry but reduced exercise tolerance [40, 41, 44]. Arterial blood gas analysis and the diffusing capacity for carbon monoxide are indicated in selected candidates with unclear hypoxia-altitude simulation test findings [45]. In the neurological domain, formal neuropsychological testing is indicated in the presence of cognitive complaints or of a relevant history, and electroencephalography is indicated for suspected or documented seizure disorder [46]. Endocrine workup includes cortisol axis testing for suspected adrenal insufficiency and thyroid function for clinical indication [47]. Ophthalmic second-tier assessment includes optical coherence tomography and automated perimetry for candidates with a history of optic nerve or macular disease [24, 25]. Hematological workup includes coagulation-factor assays for bleeding disorders and a thrombophilia panel for candidates with prior thrombosis [48, 49]. Dental workup is indicated in candidates with a history of barodontalgia during commercial aviation, recent endodontic or surgical procedures within the prior 90 days, or untreated dental caries, with pre-flight resolution of acute pathology required before clearance [50]. Civilian orbital flight has documented transient but measurable shifts in the oral microbiome during short-duration exposure, supporting the inclusion of dental indication-driven evaluation as part of the system-specific battery [51]. Specialist psychiatric evaluation follows a positive psychological screen or a documented psychiatric history [14].

Tier 3 Mission-Class-Specific Assessment

Tier 3 investigations are triggered by the mission class the participant is entering, independently of the personal medical history, and reflect the physiological and operational demands that increase with mission duration and altitude. For suborbital missions with brief microgravity exposure, centrifuge familiarization with a physiological stress component and an anxiety desensitization component constitutes the core Tier 3 investigation, and identifies participants most likely to experience acceleration-phase intolerance [14, 21, 52]. For orbital free-flyer missions of up to 5 days, centrifuge familiarization is combined with an extended cardiovascular baseline that includes 24-hour ambulatory electrocardiography and baseline optical coherence tomography [26]. For International Space Station visiting-crew missions of 10 to 20 days, the above investigations are supplemented by dental evaluation to exclude acute pathology, carbon dioxide sensitivity counseling and documentation, and a continuity plan for continuous positive airway pressure therapy where obstructive sleep apnea is present [53, 54]. Pre-flight quarantine and infection-prevention protocols modeled on the commercial implementation of the NASA Health Stabilization Program during the Crew Demo-2 mission are incorporated into the immediate prelaunch window for ISS visiting-crew missions [55], and serological baseline with post-flight surveillance for latent-virus reactivation is supported by salivary microbiome and viral-load data in career astronauts after longer-duration exposure [56]. Planned post-flight ophthalmologic follow-up at 3 and 6 months is documented at this stage for all missions of 10 days or longer, on the basis that the duration enters the boundary region for spaceflight-associated neuro-ocular syndrome [26].

Decision Pathway and Documentation

The three tiers proposed here can converge in a structured decision documenting a categorical fitness determination (fit, case-by-case evaluation, or unfit or defer; Table 4), a medical countermeasure plan that specifies pharmacological prophylaxis, supplemental oxygen provision, and device continuity as relevant, an in-flight monitoring plan calibrated to vehicle capability, an informed consent record that explicitly states the limits of current knowledge for the specific mission class, and a post-flight follow-up schedule with minimum checkpoints at the immediate post-landing interval, at 1 month for suborbital flights, and at 3 and 6 months for orbital and longer missions [57-59].

**Table 4:** Practical decision framework for pre-flight eligibility of the commercial spaceflight participant, organized by organ system. Abbreviations: FEV1, forced expiratory volume in one second; HbA1c, glycated hemoglobin; SpO₂, peripheral oxygen saturation

Organ system	Fit for flight	Case-by-case evaluation	Unfit or defer
Cardiovascular	Controlled hypertension on stable therapy; stable coronary artery disease after complete revascularization ≥12 months; paroxysmal atrial fibrillation on rate or rhythm control with adequate anticoagulation; clinically stable valvular disease without hemodynamic compromise	Prior revascularization within 12 months with documented functional recovery; implantable cardioverter-defibrillator or pacemaker with verified device stability; moderate valvular disease; left ventricular ejection fraction 40% to 50%	Decompensated heart failure; unstable coronary syndrome within 6 months; severe pulmonary hypertension; left ventricular ejection fraction < 40%; uncontrolled arrhythmia
Pulmonary	Stable asthma on controller therapy with FEV1 ≥ 80% predicted; obstructive sleep apnea on effective positive-airway-pressure therapy; mild chronic obstructive pulmonary disease with SpO₂ ≥ 95% at rest	Moderate chronic obstructive pulmonary disease with FEV1 50% to 80%; restrictive lung disease with preserved functional capacity; post-hypoxic altitude simulation test SpO₂ 92% to 95% with planned supplemental oxygen	FEV1 < 50% predicted; resting SpO₂ < 92%; severe uncontrolled asthma with recent hospital admission; active pulmonary infection
Metabolic and endocrine	Type 2 diabetes with HbA1c < 7.5% and no history of severe hypoglycemia; hypothyroidism on stable levothyroxine with normal thyrotropin; adrenal insufficiency on a documented stress-dose protocol	Insulin-dependent diabetes with continuous glucose monitoring and a mission-specific continuity plan; recently adjusted thyroid replacement regimen; glucocorticoid dependence requiring in-flight dose planning	HbA1c ≥ 9%; recurrent severe hypoglycemia; uncontrolled hyperthyroidism; untreated or unstable adrenal insufficiency
Neurological	History of isolated and fully recovered transient ischemic attack ≥ 12 months earlier; controlled migraine without complicated features; history of single unprovoked seizure with ≥ 5-year seizure-free interval off medication	Seizure disorder on stable antiepileptic therapy for ≥2 years; history of ischemic stroke with full recovery ≥12 months earlier; complicated migraine with aura	Recent stroke or transient ischemic attack within 12 months; active seizure disorder with breakthrough events; degenerative neurological disease with functional impairment
Psychiatric	Stable mood disorder on maintenance therapy; well-controlled anxiety without panic features; documented capacity to complete confined-environment training	Moderate anxiety with trigger overlap in confinement or sensory overload; remitted post-traumatic stress disorder; attention-deficit conditions on stable therapy	Active psychotic disorder; severe uncontrolled mood disorder; active substance use disorder; claustrophobia of documented disabling severity
Hematological and coagulation	Well-controlled long-term anticoagulation with stable international normalized ratio or documented direct oral anticoagulant adherence; minor clotting factor variants without bleeding history	Anticoagulation regimen recently modified; prior thromboembolic event with completed treatment course; mild bleeding disorder with documented hemostatic reserve	Active bleeding disorder; uncontrolled anticoagulation; recent major thromboembolism within 3 months
Oncological	Cancer with completed curative treatment, no evidence of disease, and ≥12 months of stable surveillance imaging; indolent hematological condition without treatment requirement	Active surveillance within 12 months of treatment completion; adjuvant endocrine therapy for breast or prostate cancer with preserved functional capacity	Active malignancy under chemotherapy or radiotherapy; metastatic disease; recent surgery with incomplete recovery
Ophthalmological	Stable refractive error corrected by glasses, contact lenses, or prior refractive surgery with ≥6-month stability; documented absence of optic disc abnormality on baseline optical coherence tomography	Prior intraocular surgery within 6 months; stable glaucoma on medical therapy; mild retinal pathology under surveillance	Active retinal detachment; uncontrolled glaucoma; recent vitreoretinal surgery within 3 months
Reproductive	Documented non-pregnant status confirmed by β-human chorionic gonadotropin on the day of flight; contraception plan in place for participants of reproductive age with flight scheduled within 12 months	Early postpartum period beyond 6 weeks with completed lactation or stable milk supply	Pregnancy at any gestational stage; active lactation intended to continue during the flight window
Infectious and immune	Up-to-date routine adult vaccination consistent with travel-medicine practice; no active infectious condition at the day-of-flight assessment	Recent minor infectious episode fully resolved within 2 weeks; borderline vaccination status with catch-up initiated	Active systemic infection; active reactivation of herpetic or other latent infection; recent vaccination with ongoing systemic reactogenicity

Implications and future directions

The evaluation routine proposed in this review rests on an evidence base that has three known limitations. The available centrifuge cohorts number fewer than two hundred self-selected volunteers assembled at a single facility, the integrated multi-omic data on civilian orbital flight come from the four participants of the Inspiration4 mission and the four of Polaris Dawn, and a sex-balanced long-term follow-up of commercial participants after return is not yet available [3, 4, 8]. The tier-based routine has therefore been constructed to be conservative at its Tier 1 baseline and permissive at its Tier 2 indication-driven component, on the assumption that indication-driven investigations can be revised as the evidence base expands without disrupting the universal baseline. A practical implication of this structure, independent of its aerospace purpose, is that the Tier 1 baseline doubles as a comprehensive adult health assessment that often identifies previously undiagnosed hypertension, dysglycemia, or paroxysmal atrial fibrillation in participants who presented for a spaceflight evaluation and leave with additional information relevant to their long-term health [10, 12]. Future research priorities include prospective enrollment of commercial participants into multi-site registries with standardized pre- and post-flight biomarker panels in the tradition of the Space Omics and Medical Atlas, sex-balanced recruitment to resolve the current male predominance in the evidence base, and longitudinal follow-up at 1, 3, and 5 years after flight to characterize delayed outcomes that short-term studies cannot capture [3, 5]. Regulatory harmonization between the 2006 FAA guidance and the evidence accumulated since then remains a separate and complementary task for the aerospace-medicine community [11]. The routine proposed here is intended as an operational starting point for commercial operators and evaluating physicians, not as a final standard, and its performance in practice can only be established through the prospective data that the expanding commercial era will generate.

## Conclusions

The commercial spaceflight era, inaugurated in 2021, has introduced a participant population whose age range, comorbidity burden, and mission profile distribution are not addressed by the foundational 2006 FAA guidance on medical screening of aerospace passengers. A pre-flight medical evaluation routine tailored to this population must accommodate an age range of 18 to 90 years, medically controlled cardiopulmonary, metabolic, and oncological comorbidities, and mission classes that span three orders of magnitude in duration and physiological demand. The three-tier framework proposed here, namely a universal baseline applied to every candidate, indication-driven investigations triggered by clinical findings or prior diagnosis, and mission-class-specific investigations calibrated to the selected vehicle and profile, provides an operational structure that can be consistently applied across commercial operators and evaluating physicians. Three limitations of the current evidence base, namely centrifuge cohorts of fewer than 200 self-selected volunteers, integrated multi-omic data from only twelve civilian orbital participants across Inspiration4, Polaris Dawn, and Fram2, and the absence of sex-balanced longitudinal post-flight follow-up, are explicit in the proposal and constrain its generalizability. The routine is therefore offered as an operational starting point rather than a final standard, and its prospective validation as the commercial era generates additional data will determine its performance in practice.
